# Supplementary material for: Cancer subtype identification using somatic mutation data
Source: Br J Cancer. 2018 May 16;118(11):1492–501. doi: 10.1038/s41416-018-0109-7 (PMC5988673; doi:10.1038/s41416-018-0109-7)
Supplement: Supplementary file 1 — Supplemental materials and methods [file 41416_2018_109_MOESM1_ESM.pdf]

SUPPLEMENTAL MATERIALS AND METHODS

Curation of clinical data

We used R package RTCGAToolbox to download clinical data for all TCGA. We used the *getFirehoseDatasets()* function to get a list of all tumors (23 when we accessed these data), and the function *getFirehoseRunningDates()* to get a list of the available Firehose dates. We prepared the clinical data for each cancer type separately. First, we checked at which of the available Firehose dates clinical data had been uploaded. We then downloaded clinical data from the first available Firehose date that included this data type for the cancer type of interest, as well as for the next available date. We made column names consistent by removing all punctuation characters and spaces from these names, and by converting all characters to lowercase. We then merged the data by keeping all data from the latest (of the two) Firehose dates, but added any patients or columns from the previous date that were not available in the data uploaded at the later date. We continued this, comparing clinical data uploaded at a particular time point to the combined set of data previously uploaded. This way, we contained as much clinical information as possible, while containing the most up-to-date entries for those data points that were uploaded multiple times (for example, follow-up data). For visualization of clinical parameters in Figure 3, we converted phenotypic parameters according to Supplemental Table 1. We merged duplicate clinical parameters, such as "years to birth" and "age at diagnosis."

Dissimilarity metrics

The Mahalanobis distance is similar to Euclidean distance except that it normalizes the data based on a covariance matrix, making the distance metric scale-invariant. Intuitively, it asks what the distance of a point in n-dimensional space is for a given n-space distribution. The binomial dissimilarity index is derived from the binomial deviance under the null hypothesis that the two compared communities are equal. For our data, this implies that the proportions of various signatures are more similar amongst similar samples.

The larger the distances or dissimilarity measures between the samples of a specific cancer, the greater the hidden structure revealed to help in differentiating samples potentially linked to phenotypic properties. The greatest median measurement and variation amongst cancer types when using the binomial dissimilarity index on the biological pathway de-sparsified mutation data, and therefore selected this metric to identify cancer-specific mutation subtypes associated with survival.
