# Supplementary material for: Cancer subtype identification using somatic mutation data
Source: Br J Cancer. 2018 May 16;118(11):1492–501. doi: 10.1038/s41416-018-0109-7 (PMC5988673; doi:10.1038/s41416-018-0109-7)
Supplement: Supplementary file 2 — Supplemental Figure 1 [file 41416_2018_109_MOESM2_ESM.pdf]

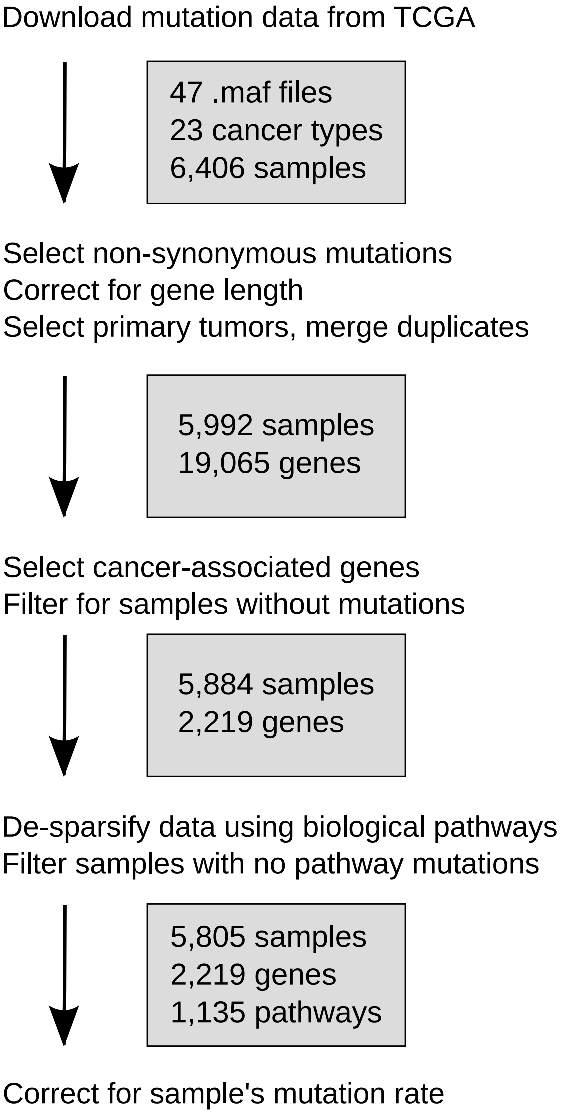
Download mutation data from TCGA
47 .maf files
23 cancer types
6,406 samples
Select non-synonymous mutations
Correct for gene length
Select primary tumors, merge duplicates
5,992 samples
19,065 genes
Select cancer-associated genes
Filter for samples without mutations
5,884 samples
2,219 genes
De-sparsify data using biological pathways
Filter samples with no pathway mutations
5,805 samples
2,219 genes
1,135 pathways
Correct for sample's mutation rate
