# Supplementary material for: Cancer subtype identification using somatic mutation data
Source: Br J Cancer. 2018 May 16;118(11):1492–501. doi: 10.1038/s41416-018-0109-7 (PMC5988673; doi:10.1038/s41416-018-0109-7)
Supplement: Supplementary file 3 — Supplemental Figure 2 [file 41416_2018_109_MOESM3_ESM.pdf]

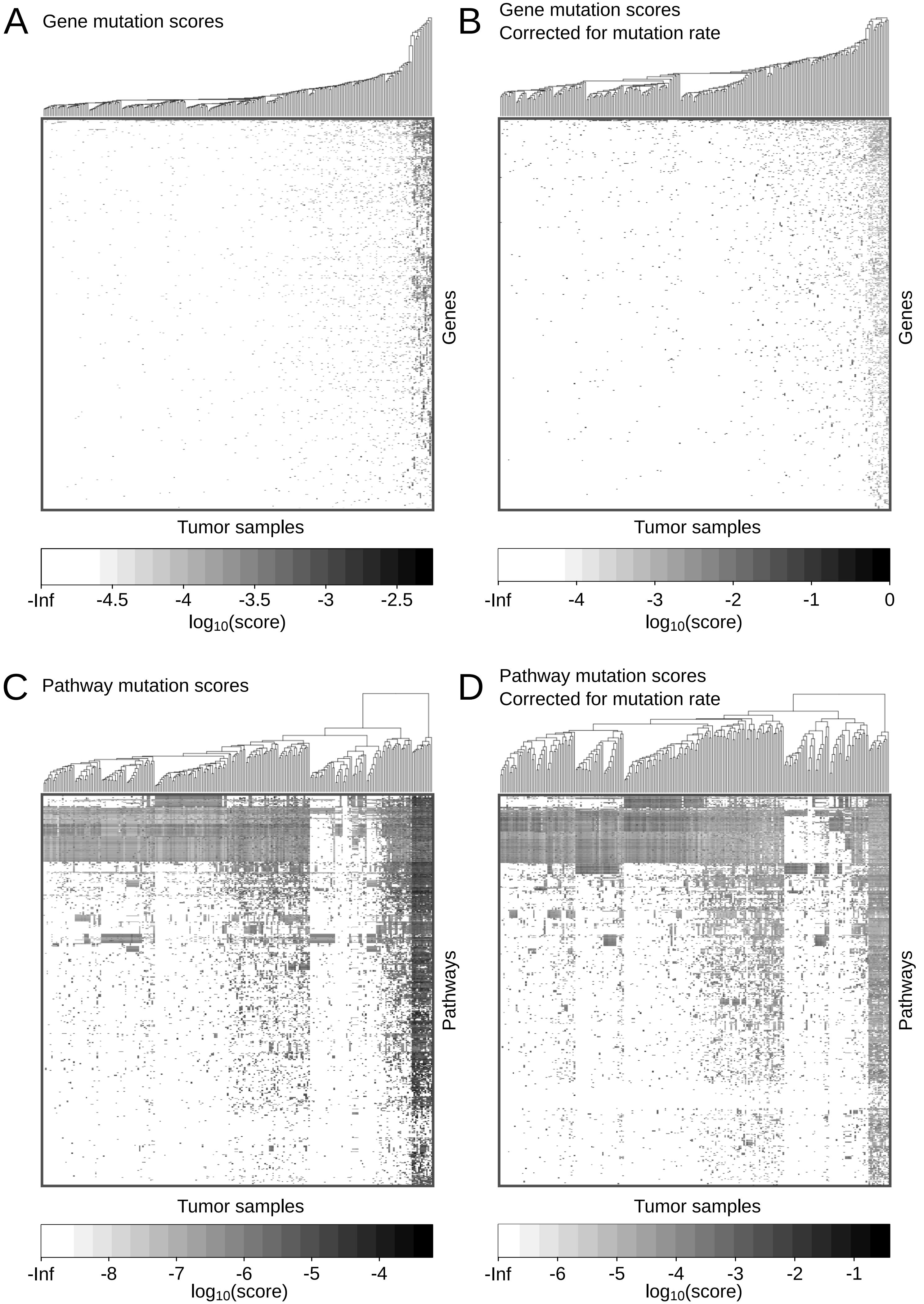

A
Gene mutation scores
Genes
Tumor samples
-Inf
-4.5
-4
-3.5
-3
-2.5
log10(score)
B
Gene mutation scores
Corrected for mutation rate
Genes
Tumor samples
-Inf
-4
-3
-2
-1
0
log10(score)
C
Pathway mutation scores
Pathways
Tumor samples
-Inf
-8
-7
-6
-5
-4
log10(score)
D
Pathway mutation scores
Corrected for mutation rate
Pathways
Tumor samples
-Inf
-6
-5
-4
-3
-2
-1
log10(score)
