# Supplementary figures and images for: Cancer subtype identification using somatic mutation data
Source: Br J Cancer. 2018 May 16;118(11):1492–501. doi: 10.1038/s41416-018-0109-7 (PMC5988673; doi:10.1038/s41416-018-0109-7)

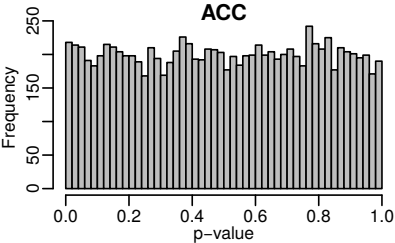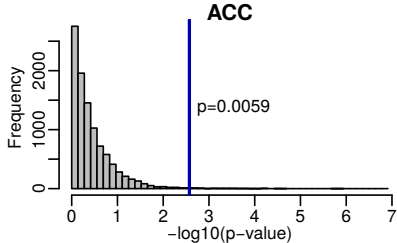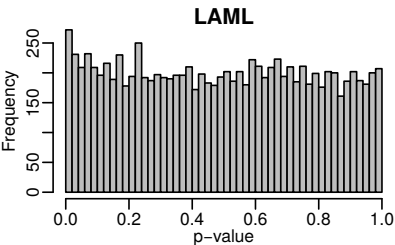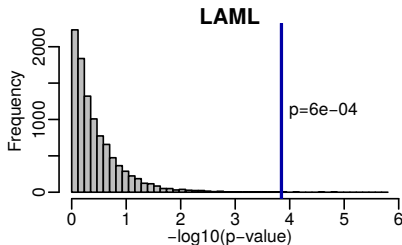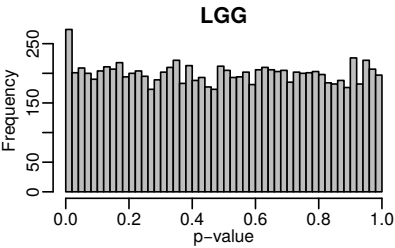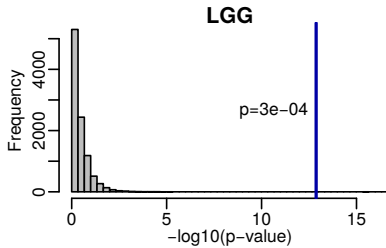

Supplement: Supplementary file 4 — Supplemental Figure 3 [file 41416_2018_109_MOESM4_ESM.pdf]

**A**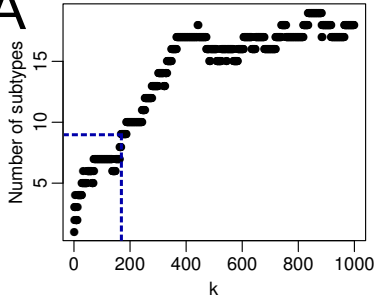**B**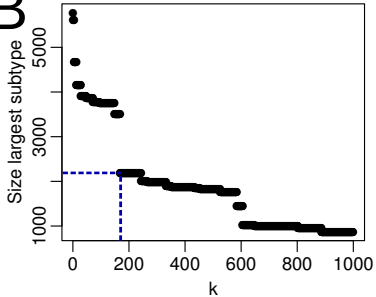

Supplement: Supplementary file 5 — Supplemental Figure 4 [file 41416_2018_109_MOESM5_ESM.pdf]

A

## Cancer types

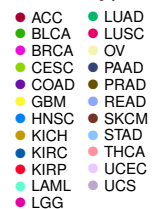

## Subtypes

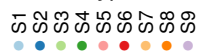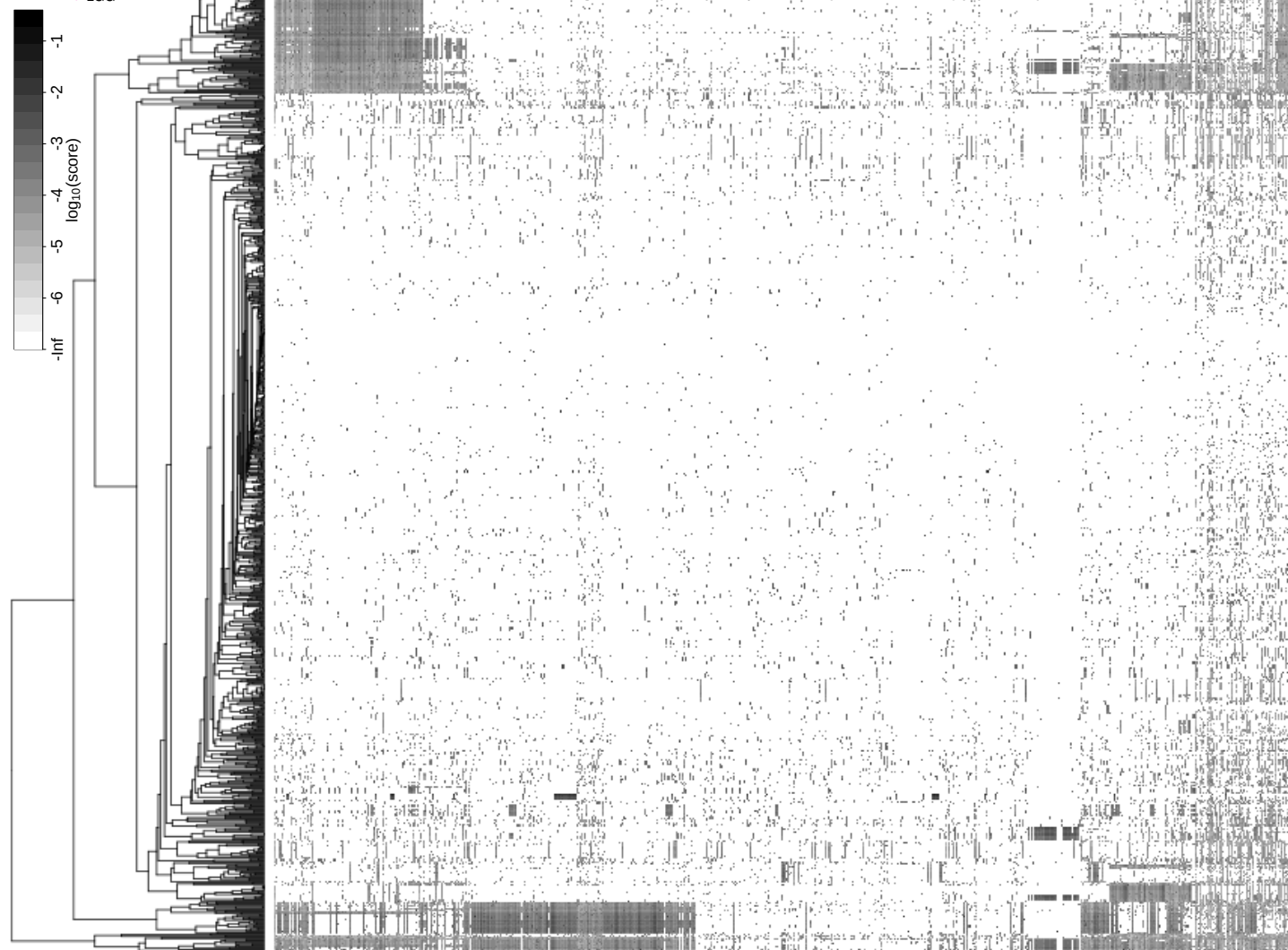

B

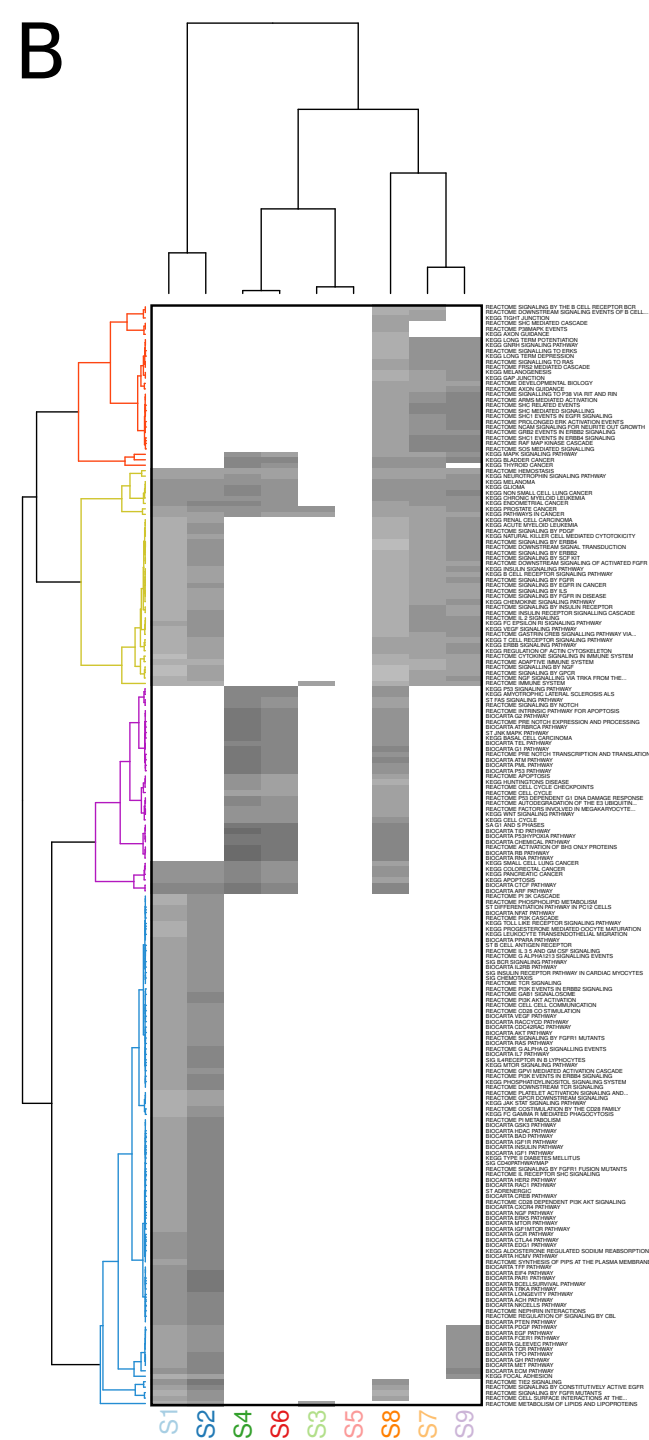

Supplement: Supplementary file 6 — Supplemental Figure 5 [file 41416_2018_109_MOESM6_ESM.pdf]
